# High-calcium milk improves osteoporosis in postmenopausal women by regulating intestinal flora and steroid hormone biosynthesis

**DOI:** 10.3389/fnut.2025.1607968

**Published:** 2025-08-06

**Authors:** Yanyan Zhao, Xianping Li, Yanpin Liu, Qishan Wang, Junying Zhao, Hang Pan, Huo Chen, Bin Liu, Weicang Qiao, Li Lin, Yue Jin, Lijun Chen

**Affiliations:** ^1^School of Public Health, Shandong Second Medical University, Weifang, China; ^2^National Engineering Research Center of Dairy Health for Maternal and Child, Beijing Sanyuan Foods Co., Ltd., Beijing, China; ^3^Shenyang Hongmei Foods Co., Shenyang, China; ^4^Key Laboratory of Dairy Science, Ministry of Education, Food Science College, Northeast Agricultural University, Harbin, China

**Keywords:** functional food, bone density, microbiome, metabolomics, postmenopausal women

## Abstract

**Background:**

Postmenopausal calcium loss increases osteoporosis risk in middle-aged and older women. While dairy products are a known calcium source that supports bone health, limited research addresses their specific effects on osteoporosis prevention in this population.

**Methods:**

A one-year randomized controlled trial recruited 97 postmenopausal women, randomly assigned to a high-calcium milk group (HCM, 51), consuming 400 mL nutrient-enriched fresh milk daily, or a control milk group (CM, 46), consuming 400 mL of regular fresh milk.

**Results:**

A one-year randomized controlled trial showed that the high-calcium milk group significantly increased lumbar spine bone mineral density (L1-4 BMD), slowed bone loss in the left hip and femoral neck, elevated serum phosphorus and 25-hydroxyvitamin D levels, and modulated the bone formation marker procollagen type I N-terminal propeptide compared with the regular milk group at 6 months. 16S ribosomal ribonucleic acid sequencing showed that high-calcium milk significantly altered the *β*-diversity of the intestinal flora, increasing the abundance of beneficial bacteria such as *Bacteroides*, *Oscillibacter*, and *Subdoligranulum*, while decreasing the abundance of Firmicutes and *Weissella* at 12 months. Metabolomics analysis revealed that high-calcium milk improved bone quality by modulating steroid hormone biosynthesis and arachidonic acid metabolic pathways, and that L1–4 BMD was positively correlated with *Faecalibacterium* spp. and adenine nucleotide.

**Conclusions:**

Our study suggests that high-calcium milk can effectively delay postmenopausal osteoporosis by regulating intestinal flora and metabolic pathways, providing a new target for osteoporosis intervention.

**Clinical trial registry number:**

ChiCTR2200064825 (https://www.chictr.org.cn/bin/home).

## 1 Introduction

Postmenopausal osteoporosis (PMO) is a metabolic bone disease caused by estrogen deficiency. It is characterized by reduced bone mineral density (BMD), increased bone fragility, and a higher risk of fractures, leading to loss of physical function, increased morbidity and mortality, and reduced quality of life in postmenopausal women (1–3). Approximately 9–38% of women over the age of 50 are affected by osteoporosis (4). With increased life expectancy, more people are at risk of fractures, placing a significant burden on public health, healthcare systems, and the economy (5). Studies have shown that low calcium intake and insufficient vitamin D are risk factors for low bone mineral content, increased hip fracture risk, and osteoporosis in women (6–8). Although calcium is found in many foods, milk and dairy products are considered the best choice for building bone tissue during growth and reducing bone mineral loss over a lifetime (9, 10). Vitamin D and casein phosphopeptides (CPP) in dairy products are known to promote calcium absorption, hydroxyapatite formation, and bone health (8). Additionally, whey proteins inhibit osteoclast activity and stimulate osteoblast proliferation and differentiation, while minerals in dairy products support osteoblast function by increasing fat metabolism and regulating calcium–phosphorus balance. Lactose also regulates the production of short-chain fatty acids (SCFAs) by gut microbes, preventing bone loss. Evidence from randomized controlled trials suggests a positive correlation between milk and dairy product intake and BMD (11). A clinical study in older women showed that combined calcium and vitamin D supplementation reduces secondary hyperparathyroidism, slows the rate of bone loss, and lowers fracture risk (12). Previous studies have also shown that dietary supplementation with calcium-fortified milk or milk powder reduces hip and spine bone loss in postmenopausal women with low dietary calcium intake (13–15).

It is now widely recognized that increasing calcium and vitamin D intake is an important healthcare strategy for preventing osteoporosis, with the most common approach being the addition of these nutrients to milk. However, current research on the role of dairy products in osteoporosis prevention primarily focuses on fermented dairy products fortified with different probiotics, powdered milk fortified with vitamin D and calcium, and ambient milk with a long shelf life. As consumers become more health-conscious, they tend to choose fresh pasteurized milk with a shorter shelf life; however, few studies have investigated its impact on osteoporosis. The study aimed to evaluate the impact of high-calcium fresh milk on bone health in postmenopausal women, particularly its effects on BMD and bone loss. By integrating data from the gut microbiome, gut metabolome, and serum metabolome, we aim to more comprehensively reveal the disease pathogenesis and evaluate intervention effects, providing new ideas and methods for clinical research.

## 2 Methods

### 2.1 Study participants

**Figure 1:**
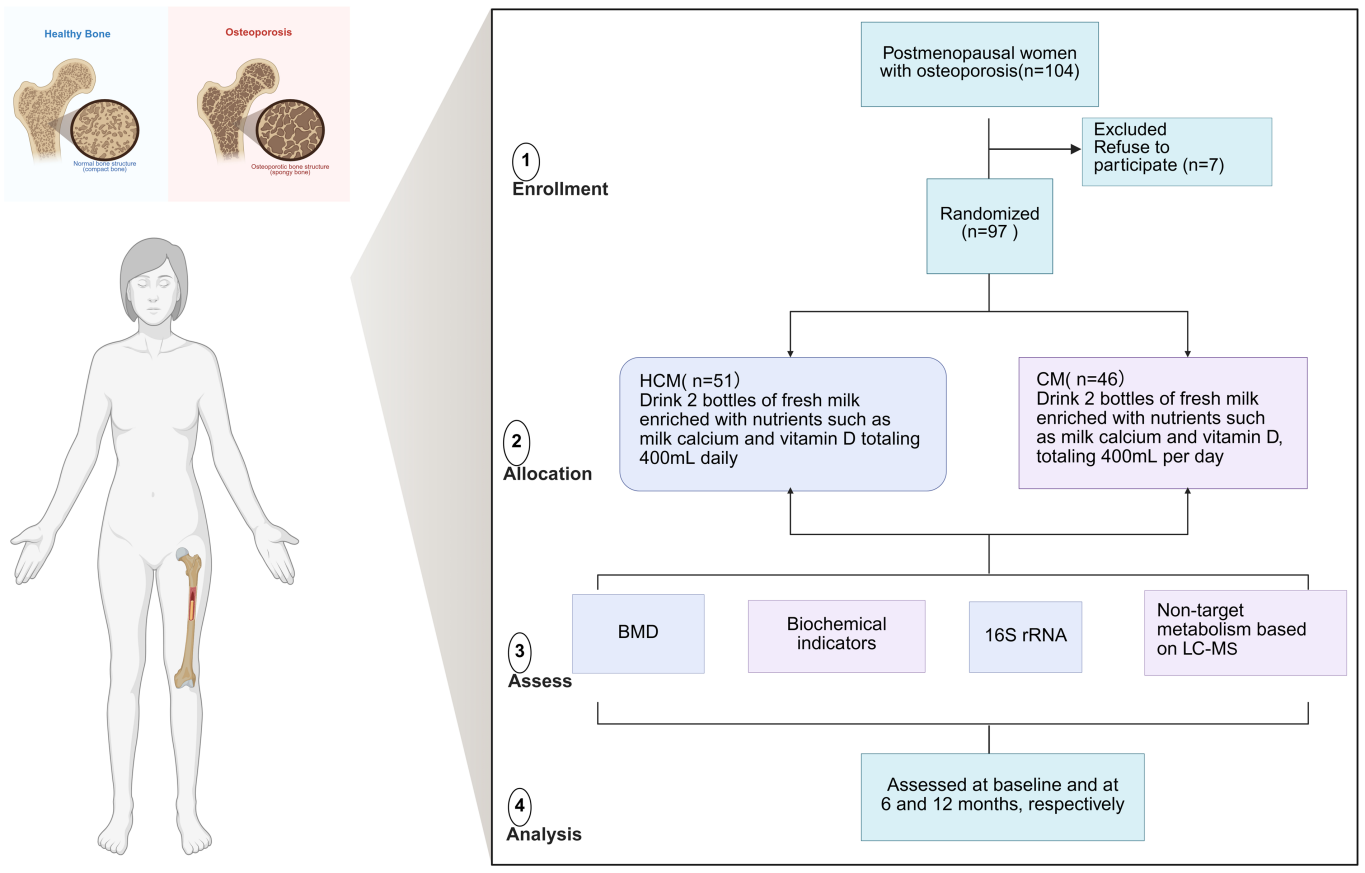
Flowchart of the dairy intervention in osteoporosis study in menopausal women. BMD, bone mineral density; CM, Control milk group; HCM, High Calcium Milk Group.

We studied 104 women, aged 48–73, who had been menopausal for over 1 year and had low bone mass or osteoporosis in the lumbar spine or femur. All participants were stable residents of urban Beijing and were recruited through the community. Exclusion criteria included a history of smoking, alcohol or coffee consumption, inability to care for oneself, lactose intolerance, psychiatric disorders, serious organic or infectious diseases, a history of osteoporotic fractures, gastrointestinal surgery, or any disease known to affect bone metabolism. Patients were also excluded if they had a history of medication use or intake of dietary supplements known to affect bone metabolism during the intervention period, including milk products, soya milk, vitamin D, vitamin K, multivitamins, and calcium tablets. All participants provided and signed informed consent prior to participation in the study. Of the 104 women enrolled, 97 completed the study, seven participants withdrew and opted for medication intervention. The clinical trial was approved by the Medical Ethics Committee of Beijing Ditan Hospital, affiliated with Capital Medical University, and the China Clinical Laboratory Centre (approval number: ChiCTR2200064825; ethical number: Beijing Dilun Ke Zi [2022] No. (031)-02) (Figure 1).

### 2.2 Research design

This 12-month nutritional intervention used a double-blind, randomized controlled design to evaluate the effects of fresh milk enriched with lacto-calcium, vitamin D, and CPP on bone health in postmenopausal women. After signing the informed consent form, participants were randomly assigned to two groups using Excel-generated random numbers:

(1) The high-calcium milk group (HCM; *n* = 51) consumed two bottles of fresh milk enriched with lactic calcium and vitamin D, totaling 400 mL daily.
(2) The control milk group (CM; *n* = 46) consumed two bottles of plain fresh milk totaling 400 mL daily (Table 1).

**Table 1:** Nutritional composition of high-calcium fresh milk and control mil.

Nutritional content (per 100 g)	High-calcium milk	Control milk
Energy, kJ	270	270
Protein, g	3.1	3.1
Fat, g	3.7	3.7
Carbohydrates, g	4.7	4.7
Sodium, mg	68	68
Vitamin D, μg	1.3	–
Calcium, mg	200	110
Casein phosphopeptide, mg	30	–

The high-calcium fresh milk contained raw cow milk, milk mineral salts, CPP, vitamin D, microcrystalline cellulose, mono- and diglyceride fatty acid esters, sodium tripolyphosphate, sodium carboxymethylcellulose, carrageenan, and xanthan gum. Participants in both groups consumed the provided milk in addition to their daily diet for 12 months and were instructed not to change their lifestyle or dietary habits during the study. Nutritional habits were assessed at baseline, 6, and 12 months. Adherence to the intervention was monitored by telephone, and questionnaires were collected every 3 months.

### 2.3 Evaluation of the effectiveness of the intervention

During the intervention period, participants in all study groups underwent scheduled examinations. These examinations were conducted at three time points: pre-intervention (baseline), 6 months, and 12 months post-intervention.

#### 2.3.1 Anthropometry and bone densitometry

Height (cm) and weight (kg) were measured at pre-intervention (baseline), 6 months, and 12 months using a standardized height and weight measuring device. Body mass index (BMI) was calculated as weight divided by the square of height (kg/m^2^). BMD of the femoral neck, total hip, and lumbar spine (L1–L4) was measured using dual-energy X-ray absorptiometry (DPX-L, Lunar Corp., Madison, WI, United States). Patients were positioned according to standard procedures for each scan, and all follow-up scans were analyzed using the scan comparison model provided by the manufacturer. All BMD scans were analyzed by the same investigator in a blinded manner.

#### 2.3.2 Biochemical measurements

Blood samples were collected at pre-intervention (baseline), 6 months, and 12 months after an overnight fast (i.e., 12 h of fasting). Serum was collected using a vacuum blood collection tube, gently mixed 4–5 times, allowed to stand at 4°C for 15–30 min, and then centrifuged at 1,200 g for 8 min. The upper layer of yellowish serum was transferred into a 5 mL centrifuge tube using a pipette, and a protease inhibitor (1, 16) was added. The mixture was thoroughly mixed and stored at −80°C. Serum bone metabolism indicators included insulin-like growth factor I (measured using an ELISA kit from R&D Systems), PINP, CTX, OC, and TRACP-5b (measured using an ELISA kit, Guangzhou Fikang Biotechnology Co., Ltd.). ALP, AST, calcium, and phosphorus levels were measured using a Beckman AU5800 automated biochemistry analyzer (United States), while 25-hydroxyvitamin D [25(OH)D] was measured using an AB SCIEX. 25(OH)D was quantified using an AB SCIEX 6500 triple quadrupole liquid chromatography-mass spectrometry (LC–MS).

### 2.4 Biospecimen testing

Participants collected fecal samples at three time points: pre-intervention (baseline), 6 months, and 12 months. Each sample, approximately 10 g, was collected in PSP tubes (Stratec, Germany), with or without protective fluid, and stored at −80°C.

Sequencing of the 16S ribosomal ribonucleic acid (rRNA) gene of intestinal flora: ① Genomic deoxyribonucleic acid (DNA) was extracted from 100 mg of fecal samples using the QIAamp Rapid Fecal DNA Mini kit (Qiagen, GmbH, Hilden, Germany). Ten nanograms of DNA were used as a template for polymerase chain reaction (PCR) amplification of the 16S rRNA gene in the V3–V4 region (primers: CCTAYGGGRBGCASCAG and GGACTACNNGGGGTATCTAAT). ② PCR products were combined in equal amounts based on their concentration, detected, and the target bands were recovered after thorough mixing. ③ The constructed libraries were quantified using Qubit and quantitative PCR, assessed for quality, and sequenced on the NovaSeq 6,000 platform using PE250 sequencing. The sequencing results were merged, quality-filtered, and demultiplexed using QIIME 1.9.0. Sequences with 97% similarity were grouped into Operational Taxonomic Units and annotated against the Greengenes 16S rRNA gene database (version 13.5). Downstream data were statistically analyzed for differences using the rank test and the non-parametric Kruskal-Wallis test, following normalization by TSS and CSS methods on the Calypso 8.84 platform. False discovery rate or Benjamini-Hochberg-corrected *p*-values were applied to assess significance using two-sided statistical tests. A p-value <0.05 was considered statistically significant, and microbial genera contents were reported as mean values.

Non-targeted metabolome analysis: ① One hundred milligrams of liquid nitrogen-milled tissue samples were placed in an Eppendorf tube, followed by the addition of 500 μL of 80% methanol aqueous solution. ② The sample was vortexed, rested in an ice bath for 5 min, and centrifuged at 15,000 g at 4°C for 20 min. ③ A portion of the supernatant was diluted with mass spectrometry-grade water to achieve a methanol content of 53%. ④ The diluted sample was centrifuged at 15,000 g at 4°C for 20 min, and the supernatant was collected for LC–MS analysis. The sample was ionized using high-performance liquid chromatography, separated according to the mass-to-charge ratio (m/e or m/z) of the ions, and then detected in a mass spectrometer to obtain molecular weight information for each ion peak. Identified metabolites from the non-targeted metabolic analysis were annotated using the Kyoto Encyclopedia of Genes and Genomes (KEGG) database. For multivariate statistical analysis, data were processed using the metabolomics software metaX and subjected to principal component analysis and partial least squares-discriminant analysis (PLS-DA) to derive the VIP values for each metabolite. For univariate analysis, each metabolite’s statistical significance (*p*-value) between groups was calculated using the *t*-test, and the fold change (FC value) was determined. Differential metabolite screening criteria were set to VIP > 1, *p* < 0.05, and FC ≥ 2 or FC ≤ 0.5.

### 2.5 Statistical analysis

Data were assessed for normality using the chi-square test and presented as mean ± standard deviation. Quantitative variables were compared between groups using *t*-tests for independent samples. Independent *t*-tests were used for baseline comparisons between groups, while the Mann–Whitney test was used for non-normally distributed variables. Differences in dairy effects across measurement time points were analyzed using one-way repeated measures analysis of variance (ANOVA), followed by Tukey’s honestly significant difference *post hoc* test for within-group comparisons. Significant differences due to consumption of specific dairy products or longitudinal effects were analyzed using two-way repeated measures ANOVA or paired *t*-tests, with Tukey’s post hoc test applied as needed. For non-normally distributed variables, Wilcoxon tests and Kruskal-Wallis tests were used for within-group and between-group analyses, respectively. When significant differences were found in between-group comparisons, independent t-tests or Mann–Whitney U tests were used to identify specific time points with group differences. Statistical analyses were performed using Jamovi software (version 2.4.11), with *p* < 0.05 considered statistically significant.

## 3 Results

### 3.1 Baseline characteristics

Of the 104 women enrolled, 97 completed the study, resulting in a dropout of 7 participants. The number of participants with complete baseline and follow-up data was 51 in the HCM and 46 in the CM. These participants had a mean age of 61.2 ± 4.90 years and a mean BMI of 24.8 ± 3.06 kg/m^2^. All patients were diagnosed with either osteopenia or osteoporosis according to hospital diagnosis. Table 2 summarizes the baseline characteristics of the 97 participants who provided complete data at both baseline and follow-up examinations. No significant differences were observed between the two groups in any baseline characteristics, suggesting homogeneity in demographic, anthropometric, and body composition variables.

**Table 2:** Baseline characteristics of the study groups.

Characteristic	HCM	CM	t/U	*p*-value
Age, years	61.2 ± 5.00	61.3 ± 4.83	0.03	0.974
Height, cm	159.4 ± 4.79	159.5 ± 5.00	0.08	0.941
Weight, kg	62.8 ± 8.18	63.7 ± 10.09	0.45	0.657
BMI, kg/m^2^	24.7 ± 2.77	25.0 ± 3.39	0.45	0.655
Duration of menopause	11.0 ± 5.77	11.5 ± 6.18	0.47	0.639
L1-4BMD, g/cm^2^	0.981 ± 0.123	0.935 ± 0.107	−1.93	0.057
Left femoral neck, g/cm^2^	0.776 ± 0.130	0.777 ± 0.092	1,063	0.429
Left hip, g/cm^2^	0.856 ± 0.128	0.849 ± 0.096	1,042	0.344
Right femoral neck, g/cm^2^	0.777 ± 0.080	0.777 ± 0.085	−0.01	0.992
Right hip, g/cm^2^	0.861 ± 0.080	0.849 ± 0.101	−0.76	0.450
AST, U/L	20.61 ± 5.773	23.07 ± 9.546	1,050	0.357
Calcium, mmol/L	2.35 ± 0.192	2.34 ± 0.196	1,125	0.731
25(OH)D, ng/mL	15.64 ± 5.875	15.26 ± 4.426	1,173	1.000
TRACP-5b, U/L	1.80 ± 0.504	1.93 ± 0.607	1,082	0.513
ALP, U/L	67.65 ± 16.876	72.54 ± 20.751	1,061	0.481
P, mmol/L	1.07 ± 0.123	1.06 ± 0.146	1,125	0.729
CTX, ng/mL	0.37 ± 0.297	0.36 ± 0.257	1,165	0.954
PINP, ng/mL	84.67 ± 48.256	91.38 ± 36.629	960	0.125
OC, ng/mL	22.89 ± 8.504	23.64 ± 8.799	1,118	0.691
IGF-1, ng/mL	10.26 ± 6.232	10.14 ± 5.64	1,158	0.914

Data are presented as mean ± standard deviation (SD). The p-values were based on t-test and Mann–Whitney U test. 25(OH)D, 25 hydroxyvitamin D; ALP, alkaline phosphatase; AST, aminotransferase; BMI, body mass index; CM, control milk group; CTX, type I procollagen C-peptide; HCM, high-calcium milk group; IGF, insulin-like growth factor 1; L1-4, spine; OC, osteocalcin; PINP, type I procollagen N-terminal prepeptide; TRACP-5b, human tartrate-resistant acid phosphatase 5b.

### 3.2 Changes in BMD

**Figure 2:**
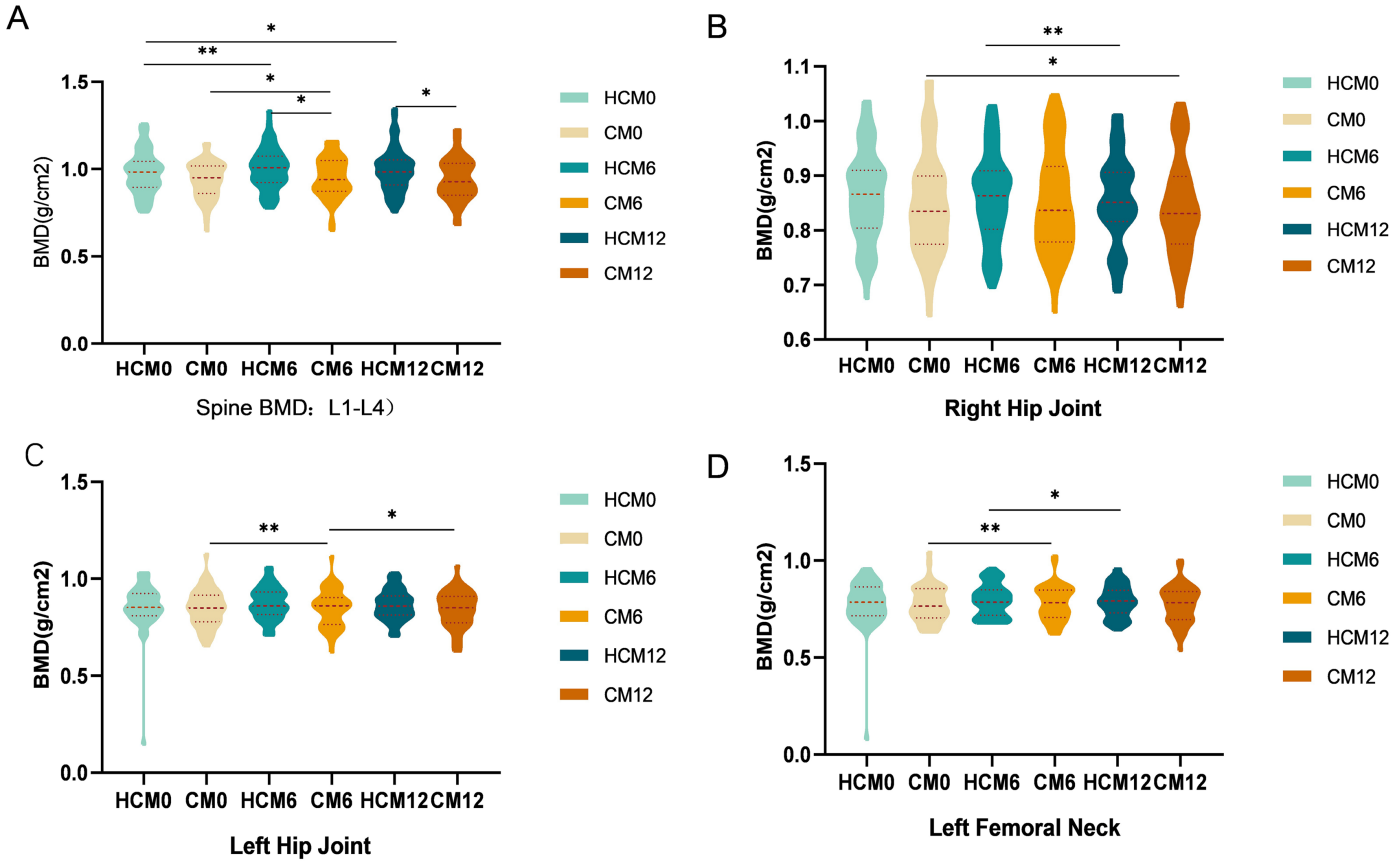
Changes in BMD during the intervention. **(A)** Spinal BMD. **(B)** Right hip BMD. **(B)** Left hip BMD. **(D)** Left femoral neck BMD. CM0, control milk group at pre-intervention; CM6, control milk group at 6 months; CM12, control milk group at 12 months; HCM0, high-calcium milk group at pre-intervention HCM6, high-calcium milk group at 6 months; HCM12, high-calcium milk group at 12 months. *p < 0.05; **p < 0.01; ***p < 0.001.

From baseline to 12 months post-intervention, high-calcium milk had a significant positive effect on BMD across all skeletal sites. In the lumbar spine, at 6 months post-intervention, L1–4 BMD increased by 2.3% in the HCM group (*p* = 0.003), compared to an increase of 1.5% (*p* = 0.017) in the CM group, resulting in a significant difference between groups (*p* = 0.035) (Figure 2A). By 12 months, L1–4 BMD in the HCM group remained significantly higher than that in the CM group (*p* = 0.041). For the total hip (Figures 2B,C), left hip BMD in the HCM group increased by 2.2% after 6 months (*p* = 0.014) and by 1.1% after 12 months, while left hip BMD in the CM group decreased by 0.1 and 0.7% at the respective time points. Both groups showed a slight decrease in right hip BMD by 12 months. At the femoral neck (Figure 2D), the left femoral neck BMD increased by 2.6% in the HCM group (*p* = 0.007) and by 0.5% in the CM group after 6 months. However, after 12 months, BMD in both groups decreased relative to 6-month values: by 0.5% in the HCM group and by 1.0% in the CM group (*p* = 0.046). No significant change in BMD was observed in the right femoral neck throughout the study.

### 3.3 Changes in biochemical indicators

Changes in serum biochemical indices in the HCM and CM groups are presented in Table 3. In the HCM, serum AST levels significantly increased by 16.4% after 6 months of high-calcium fresh milk consumption (*p* = 0.024), while no statistically significant changes were observed in the CM during this period. At 12 months of intervention, both HCM and CM groups showed reductions in serum AST levels compared to 6-month measurements, with decreases of 18.6% (*p* = 0.001) and 17.2% (*p* = 0.044), respectively, bringing levels below baseline. Blood calcium levels in the HCM group showed a significant increase at 6 months compared to those in the CM group. After 6 months of intervention, blood calcium levels significantly increased by 3.8% in the CM group (*p* = 0.014) and by 4.3% in the HCM group (*p* < 0.001). At 12 months of intervention, blood calcium levels increased significantly by 5.1% from baseline in the HCM group (*p* < 0.001), while levels significantly increased by 4.7% in the CM group (*p* = 0.003). After 6 months of intervention, 25(OH)D levels were significantly higher in both groups, with a 54.7% increase in the HCM group (*p* < 0.001) compared to a 23.6% increase in the CM group (*p* < 0.001), showing a significant difference between the groups (*p* < 0.001). At 12 months of intervention, 25(OH)D levels in the HCM group remained significantly higher than baseline, increasing by 52.6% (*p* < 0.001), whereas levels in the CM group increased by only 11.9%, which was not statistically significant. However, the difference between the two groups remained significant (*p* < 0.001). Table 4 shows *Z*-values of −4.390 and −3.408 at 6 months and 12 months, respectively, with both *p* < 0.0001, indicating that high-calcium milk significantly improved 25(OH)D levels compared to regular milk. TRACP-5b levels were significantly higher in both groups after 6 months of intervention, with a 24.4% increase in the HCM group (*p* = 0.007) and a 30.6% increase in the CM group (*p* < 0.001). After 12 months of intervention, TRACP-5b levels significantly decreased from baseline by 41.1% in the HCM group and 45.1% in the CM group (both *p* < 0.001). Compared to the 6-month levels, TRACP-5b levels were further reduced by 52.7% in the HCM group and 57.9% in the CM (both *p* < 0.001). Additionally, phosphorus levels and the bone formation marker PINP were significantly different between the two groups of postmenopausal women after 6 months of intervention (*p* = 0.046 and *p* = 0.001, respectively). PINP is a highly sensitive marker for predicting osteoporosis onset, assessing bone mass, and monitoring anti-osteoporosis therapy efficacy. At baseline, PINP levels in both HCM and CM groups were above the normal range. After 6 months of intervention, PINP levels decreased in both groups, with levels in the HCM group dropping to within the normal range (reference range of PINP: female, 31.7–70.7 ng/mL; mean, 21–78 μg/L via enzyme-linked immunoassay).

**Table 3:** Changes in biochemical indicators during the intervention.

Indicators	0 m	6 m	12 m	P-value
AST, U/L				
HCM	20.61 ± 5.773^1^	24.00 ± 6.930^2^	19.53 ± 6.067^1^	<0.001
CM	23.07 ± 9.546	27.04 ± 14.712^1^	22.39 ± 9.968^2^	0.054
*P*-value	0.375	0.756	0.266	
Calcium, mmol/L				
HCM	2.35 ± 0.192^1^	2.45 ± 0.067^2^	2.47 ± 0.132^2^	<0.001
CM	2.34 ± 0.196^1^	2.43 ± 0.066^2^	2.45 ± 0.075^2^	0.002
*P*-value	0.731	0.251	0.106	
25(OH)D, ng/mL				
HCM	15.64 ± 5.875^1^	24.20 ± 6.277^2^	23.86 ± 6.748^2^	<0.001
CM	15.26 ± 4.426^1^	18.86 ± 4.276^2^	17.08 ± 6.039	<0.001
*P*-value	1.00	<0.001	<0.001	
TRACP-5b, U/L				
HCM	1.80 ± 0.504^1^	2.24 ± 0.731^2^	1.06 ± 0.283^3^	<0.001
CM	1.93 ± 0.607^1^	2.52 ± 0.878^2^	1.06 ± 0.306^3^	<0.001
*P*-value	0.513	0.168	0.905	
ALP, U/L				
HCM	67.65 ± 16.876	67.02 ± 14.12	-	0.710
CM	72.54 ± 20.751	72.96 ± 17.624	-	0.711
*P*-value	0.418	0.071	-	
Phosphorus, mmol/L				
HCM	1.07 ± 0.123	1.07 ± 0.091	-	0.893
CM	1.06 ± 0.146	1.03 ± 0.107	-	0.229
P-value	0.729	0.046	-	
CTX, ng/mL				
HCM	0.37 ± 0.297	0.28 ± 0.200	-	0.194
CM	0.36 ± 0.257	0.34 ± 0.198	-	0.867
*P*-value	0.954	0.092	-	
PINP, ng/mL				
HCM	84.70 ± 48.256	66.26 ± 37.53	-	0.017
CM	91.38 ± 36.629	88.04 ± 38.08	-	0.782
*P*-value	0.125	0.001	-	
OC, ng/mL				
HCM	22.89 ± 8.504	22.15 ± 8.577	-	0.789
CM	23.64 ± 8.799	23.36 ± 7.78	-	0.941
*P*-value	0.691	0.448	-	
IGF-1, ng/mL				
HCM	10.26 ± 6.232	7.97 ± 4.93	-	0.068
CM	10.14 ± 5.640	9.47 ± 5.31	-	0.492
*P*-value	0.914	0.114	-	

25(OH)D, 25 hydroxyvitamin D; ALP, alkaline phosphatase; AST, alanine transaminase; CTX, type I procollagen C-terminal prepeptide; IGF-, insulin-like growth factor 1; OC, osteocalcin; PINP, type I procollagen N-terminal prepeptide; TRACP-5b, human anti-tartrate acid phosphatase 5b. ^123^Same number indicates a non-significant difference; different numbers indicate a significant difference.

**Table 4:** Effects of different dairy interventions on 25(OH)D.

Statistic	0 m		6 m		12 m	
	HCM	CM	HCM	CM	HCM	CM
Normal^1^	11	9	41	17	33	14
Insufficient^2^	23	25	10	27	17	24
Deficient^3^	17	12	0	2	1	6
*Z*-value	−0.401		−4.390		−3.408	
*P*-value^4^	0.688		0.0001		0.001	

^1^Normal indicates 25(OH)D ≥ 20 ng/mL. ^2^Insufficient indicates 12 < 25(OH)D < 20 ng/mL. ^3^Deficient, 25(OH)D < 12 ng/mL. ^4^*p*-values were based on a single-ordered list chi-square test. CM, control milk group; HCM, high-calcium milk group.

### 3.4 Intestinal flora

**Figure 3:**
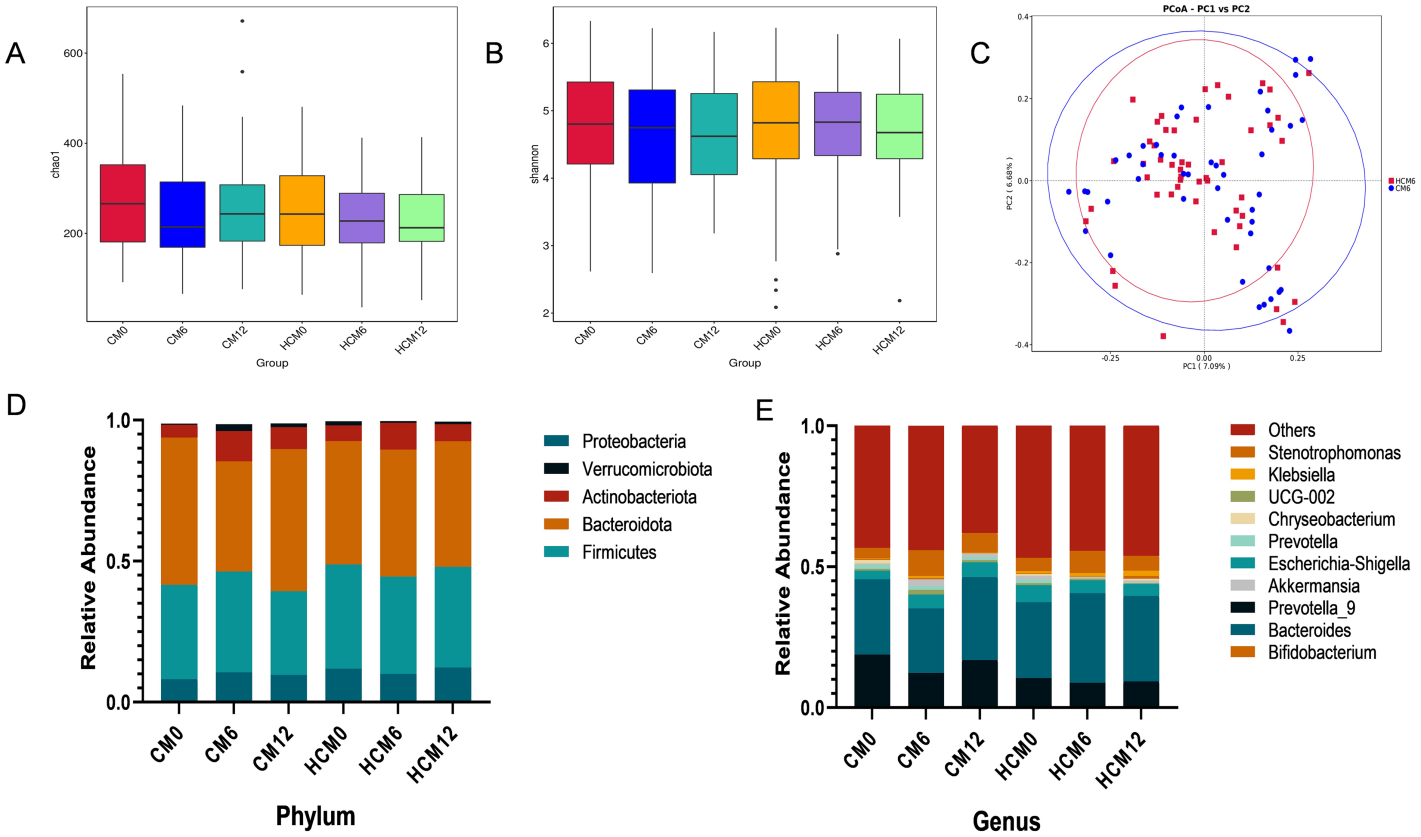
Changes in the gut flora of menopausal women with osteoporosis. **(A)** Chao1 index of microbial community richness. **(B)** Shannon index of diversity. **(C)** Principal Coordinate Analysis (PCoA) based on the Bray-Curtis distance metric at 6 months of intervention. **(D)** Relative abundance of microbial taxa at the phylum level. **(E)** Relative abundance of microbial taxa at genus level.

High-throughput sequencing of the V3–V4 region of the 16S rRNA gene was performed, and *α*- and *β*-diversity indices were calculated to assess gut microbiota structure in menopausal women with osteoporosis. Analysis of α-diversity indices showed that the gut microflora structure was similar between the two groups at enrolment (ANOVA, *p* > 0.05). The Chao1 and Shannon indices (Figures 3A,B), which reflect microbial abundance and diversity, did not differ between groups before, during, and post-intervention. After 6 and 12 months of intervention, no significant differences were observed in the Chao1 or Shannon indices between or within groups. The structure of the intestinal microbial community exhibited *β*-diversity, and principal coordinate analysis based on Bray-Curtis distance metrics (Figure 3C) showed a distinct separation in the spatial distribution of microbial communities between HCM and CM after 6 months of intervention. Principal components PC1 and PC2 explained 7.09 and 6.68% of the microbial community structure variation, respectively. The Wilcoxon test further confirmed a significant difference in microbial community structure between the HCM and CM after 6 months of intervention (*p* < 0.001).

**Figure 4:**
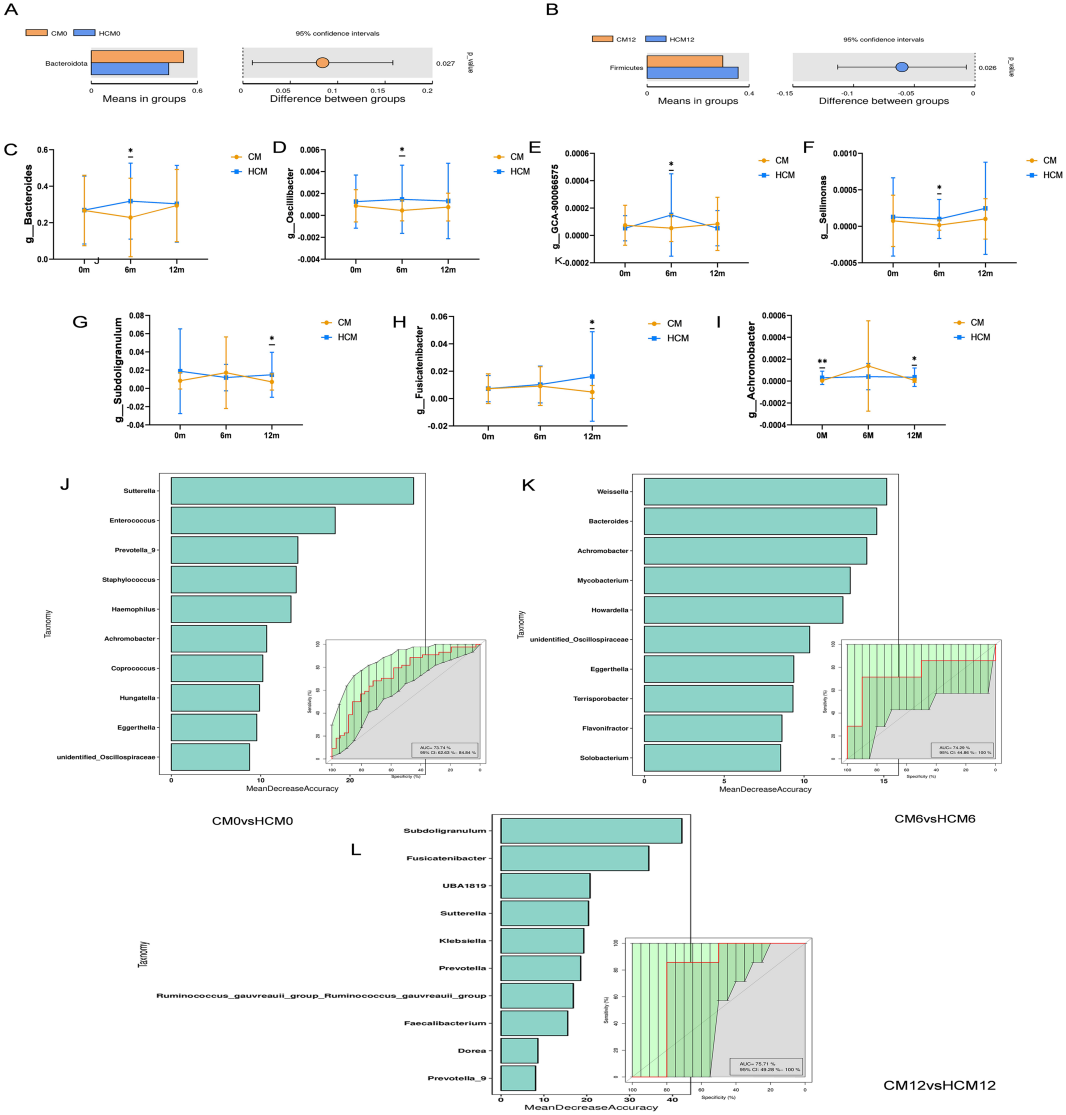
Intervention differences in bacteria between high-calcium milk and control milk. **(A,B)** Abundances of species differing at the phylum level. **(C–I)** Abundances of species differing at the genus level at different stages. **(J–L)** Random forest plots based on species abundance at different stages. *p < 0.05; **p < 0.01; ***p < 0.001.

Microorganisms were annotated across different taxonomic levels, and the five most abundant taxa at the phylum level and the 10 most abundant taxa at the genus level were selected for each group to create a bar-cumulative plot of taxa relative abundances. At the phylum level (Figure 3D), fecal sample compositions before and after the intervention showed that both groups mainly comprised the dominant phyla Bacteroidetes, Firmicutes, and Proteobacteria, which together accounted for approximately 90% of the total gut microbiome’s relative abundance. At 6 months of intervention, the relative abundance of Actinobacteria increased in both groups, while the relative abundances of Bacteroidetes and Verrucomicrobiota decreased in both groups. Conversely, the relative abundances of Firmicutes and Proteobacteria decreased in the HCM and increased in the CM. Notably, the Firmicutes-to-Bacteroidetes ratio decreased from 0.843 to 0.769 in the HCM group at baseline, whereas it increased from 0.641 to 0.916 in the CM group. By 12 months, both groups showed increased relative abundances of Actinobacteria and Proteobacteria, while Firmicutes abundance decreased. Bacteroidetes abundance increased in the HCM group but decreased in the CM group. At the genus level (Figure 3E), *Bacteroides*, *Prevotella_9*, *Bifidobacterium*, and *Escherichia-Shigella* had average relative abundances above 5%. At 6 months of intervention, the relative abundance of *Bifidobacterium* increased in both groups, while that of *Prevotella_9* decreased. In the HCM group, Bacteroides abundance increased and *Escherichia-Shigella* abundance decreased, while the opposite was observed in the CM group. At 12 months of intervention, both groups exhibited increased relative abundances of *Bacteroides* and *Bifidobacterium,* and decreased abundances of *Prevotella_9*. *Escherichia-Shigella* abundance continued to decline in the HCM group but increased in the CM group. Hypothesis testing of species abundance data between groups was conducted using the MetagenomeSeq method to obtain *p*-values, which identified species with significant differences between groups. Box plots were then generated to illustrate the distribution of species abundances between groups. At the phylum level (Figures 4A,B), the baseline abundance of Bacteroidota in the CM group was 0.522 ± 0.185, significantly higher than in the HCM group (0.438 ± 0.182, *p* = 0.026). After 6 months of intervention, Bacteroidota abundance in the CM group decreased to 0.391 ± 0.262, while it increased in the HCM to 0.450 ± 0.197, eliminating the initial difference between the two groups. For Firmicutes, abundance in the CM group decreased from 0.335 ± 0.143 to 0.296 ± 0.119, while in the HCM group, it decreased from 0.369 ± 0.170 to 0.357 ± 0.143, with both groups showing a significant reduction (*p* = 0.001). At the genus level (Figures 4C–I), *Staphylococcus* (*p* = 0.022), *Pyramidobacter* (*p* = 0.034), and *Achromobacter* (*p* = 0.005) were significantly more abundant in the HCM group than in the CM at baseline. At 6 months of intervention, *Bacteroides* (*p* = 0.042), *Oscillibacter* (*p* = 0.032), GCA-900066575 (*p* = 0.037), and *Sellimonas* (*p* = 0.036) were significantly more abundant in the HCM group than in the CM group, whereas *Weissella* abundance was significantly lower in the HCM group (*p* = 0.049). At 12 months of intervention, *Subdoligranulum* (*p* = 0.038), *Fusicatenibacter* (*p* = 0.018), and *Achromobacter* (*p* = 0.021) remained significantly higher in the HCM group than in the CM group.

To analyze species abundance using random forests, varying numbers of species were selected across different taxonomic levels using a gradient approach. Random forest models were then constructed, and receiver operating characteristic curves were plotted. Cross-validation (10-fold) was performed for each model, with key species identified based on MeanDecreaseAccuracy and MeanDecreaseGini scores. According to the random forest models at different stages, 25 species were identified as the most discriminative for assessing the effects of dairy interventions (Figures 4J–L). *Bacteroides*, *Oscillibacter*, *Subdoligranulum*, *Fusicatenibacter*, *Achromobacter*, and *Weissella*, which showed significant differences between the two groups, were identified as key species.

### 3.5 Serum metabolome

**Figure 5:**
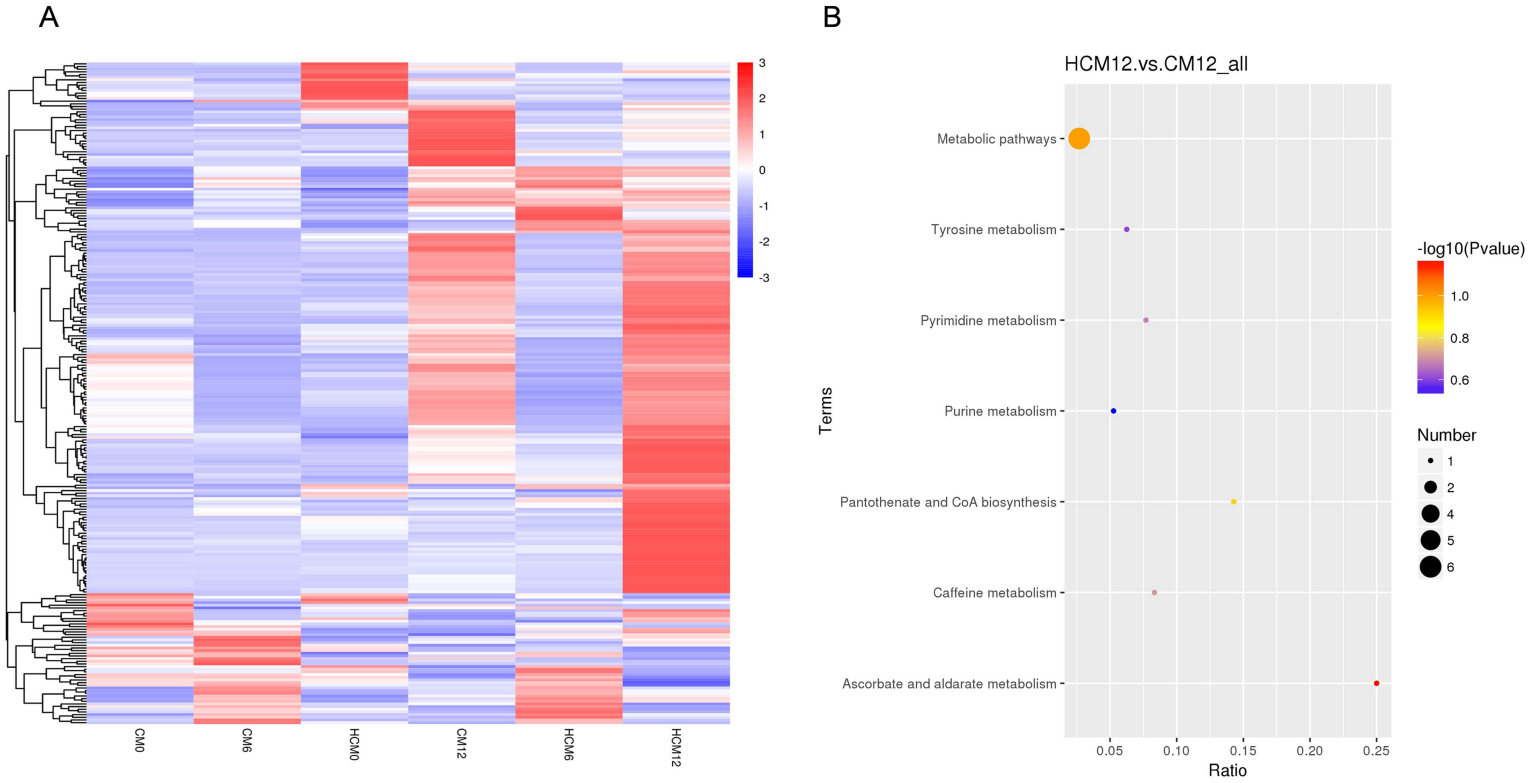
Serum differential metabolites and pathways between high-calcium milk and control milk groups. **(A)** Heat map of differential metabolites in the high-calcium milk group and the control milk group at different intervention periods. **(B)** KEGG pathway enrichment analysis after 12 months of intervention in the high-calcium milk group and the control milk group. The colors of the dots represent the *p*-values of the hypergeometric test, and the sizes of the dots represent the number of differential metabolites in the corresponding pathways.

Differential metabolites were identified using three criteria: VIP > 1.0, FC > 1.2, or FC < 0.833, and *p* < 0.05. Analysis of the differential metabolites between CM and HCM in positive and negative ion modes across different intervention periods (Figure 5A) identified 52 significant differential metabolites, with 39 upregulated and 13 downregulated (Table 5). At baseline, 13 significant differential metabolites were identified, with six upregulated and seven downregulated. After 6 months of intervention, three significant differential metabolites were identified, all of which were upregulated. After 12 months of intervention, 35 significant differential metabolites were identified, with 32 upregulated and 3 downregulated.

**Table 5:** Metabolite analysis of serum significant differences between HCM and CM at different periods.

Number	Metabolite name	Phase	RT/min	m/z	VIP	P	FC	Variation
1	2-{[methyl(2,3,4,5,6-pentahydroxyhexyl)amino]methylidene}malononitrile	0 m	7.357	289.1542	5.4664	1.6471	2.7819	↑
2	APH	0 m	7.024	346.14487	3.4045	6.5421	0.5171	↓
3	Sepiapterin	0 m	5.905	238.09	2.2726	4.1506	0.5930	↓
4	N-Methyldioctylamine	0 m	7.061	256.2997	4.0132	7.7551	0.4783	↓
5	3-(3,4-dimethylphenyl)-3,4-dihydro-1,2,3-benzotriazin-4-one	0 m	6.674	274.09057	2.9421	0.0009	0.4769	↓
6	PC 21:1_44:12	0 m	9.127	1170.876	1.6539	0.0051	0.6412	↓
7	FAHFA 20:3/22:5	0 m	10.148	633.4879	1.4644	0.0165	1.5873	↑
8	2-Hydroxycaproic acid	0 m	5.65	131.07051	1.7378	0.0252	6.8768	↑
9	PG O-12:0_20:3	0 m	10.151	701.47586	1.3379	0.0325	1.7386	↑
10	Naringenin	0 m	5.503	273.07604	1.5038	0.0369	2.3715	↑
11	FAHFA 20:3/20:2	0 m	10.161	611.50583	1.0984	0.0420	2.0383	↑
12	11-keto Testosterone (CRM)	0 m	6.354	347.18719	1.6963	0.0418	0.1582	↓
13	Equol	0 m	6.472	241.09028	2.5049	0.0360	4.4249	↓
14	L-Arabinitol	6 m	1.298	175.05797	2.2399	0.0070	1.5475	↓
15	LPC O-16:1	6 m	9.464	480.34507	2.0262	0.0369	2.8923	↑
16	PC 16:0_16:1	6 m	10.971	776.54801	2.3713	0.0387	1.5638	↑
17	2-Arachidonoyl glycerol	12 m	9.372	379.2844	3.7679	1.6701	3.5535	↑
18	2-(14,15-Epoxyeicosatrienoyl) glycerol	12 m	8.849	399.25085	3.4427	3.9848	5.395	↑
19	2,4-dihydroxyheptadec-16-en-1-yl acetate	12 m	9.16	351.25078	3.3860	0.0000	2.7276	↑
20	All-Trans-13,14-Dihydroretinol	12 m	9.768	289.25291	3.4171	0.0001	2.2489	↑
21	Monoolein	12 m	10.039	357.30001	2.5181	0.0001	1.9690	↑
22	APH	12 m	7.024	346.14487	2.5072	0.0003	1.8188	↑
23	Corticosterone	12 m	6.499	347.22148	2.0487	0.0006	1.6790	↑
24	8-Isoprostaglandin E2	12 m	5.853	351.21826	1.5437	0.0014	1.6661	↑
24	Wogonin	12 m	5.866	285.07605	2.4815	0.0036	2.4719	↑
26	Ceftibuten	12 m	6.573	393.02914	2.2561	0.0037	1.5359	↑
27	(2S)-4-Oxo-2-phenyl-3,4-dihydro-2H-chromen-7-yl beta-D-glucopyranoside	12 m	6.956	447.13511	1.8899	0.0066	2.1341	↑
28	LPE 18:3	12 m	8.334	474.26298	1.8881	0.0078	1.5199	↑
19	(5S)-5-hydroxy-1,7-diphenylheptan-3-one	12 m	8.201	305.15155	2.2920	0.0107	1.6570	↑
30	Adenin	12 m	1.382	136.06214	2.5334	0.0110	0.4518	↓
31	Palmitoyl ethanolamide	12 m	6.525	300.28985	2.0046	0.0122	2.1322	↑
32	4-(4-nitrophenylazo)aniline	12 m	5.254	265.06841	2.2393	0.0139	1.9160	↑
33	indoline-2-carboxylic acid	12 m	5.48	186.05311	1.6461	0.0156	0.4260	↓
34	7-alpha-carboxy-17-alpha-carboxyethylandrostan lactone phenyl	12 m	7.507	455.27643	1.8948	0.0160	1.7654	↑
35	L-Cystine	12 m	1.25	241.03107	1.7963	0.0191	1.5708	↑
36	3-hydroxy-N-(1-hydroxy-4-methylpentan-2-yl)-5-oxo-6-phenylhexanamide	12 m	6.95	304.19107	1.8160	0.0198	1.5793	↑
37	17α-Hydroxypregnenolone	12 m	8.109	331.2294	1.5285	0.0213	3.3654	↑
38	Sakuranin	12 m	6.701	471.13053	1.5757	0.0226	2.0277	↑
39	pyridyl]thio}prop-2-en-1-one	12 m	7.113	310.05343	1.8006	0.0276	2.6813	↑
40	Galangin	12 m	5.875	271.06023	1.8491	0.0306	3.2076	↑
41	Glycitein	12 m	6.244	285.0759	1.8252	0.0313	2.3872	↑
42	Taurine	12 m	1.386	126.02244	1.6562	0.0314	1.9914	↑
43	Cinchophen	12 m	6.025	272.06812	1.2970	0.0333	2.1881	↑
44	3,4,10,11-tetramethoxy-7,8,12b,13-tetrahydro-5H-6-azatetraphene	12 m	5.926	378.16724	1.8829	0.0338	1.5532	↑
45	6,7,8-trimethoxy-2-[3-(trifluoromethyl)phenyl]-4H-3,1-benzoxazin-4-one	12 m	5.707	382.09197	1.8106	0.0361	3.1886	↑
46	2-Methoxyestrone	12 m	6.948	299.16546	1.8372	0.0363	2.0556	↑
47	2-hydroxy-6-[(8Z,11Z)-pentadeca-8,11,14-trien-1-yl]benzoic acid	12 m	9.185	325.21403	1.6549	0.0394	3.0844	↑
48	(R)-3-Hydroxy myristic acid	12 m	7.626	243.19672	1.9194	0.0455	0.1508	↓
49	di(3-pyridyl)5-(tert-butyl)isophthalate	12 m	6.041	377.15269	1.3818	0.0466	1.6678	↑
50	2-{[(4,5-dimethoxy-2-nitrophenethyl)imino]methyl}phenol	12 m	6.181	331.12634	1.2213	0.0469	1.6793	↑
51	Genistein	12 m	5.917	269.04624	2.0086	0.0484	5.1954	↑

0 m, pre-intervention; 6 m, 6 months of intervention; 12 m, 12 months of intervention; ↓, down-regulation; ↑, up-regulation; m/z, mass-to-charge ratio; RT[min], retention time.

Based on the available metabolite annotations, after 6 and 12 months of intervention, the following metabolites were elevated in the lipid and lipid-like molecule classes in the HCM group compared to the CM group: 2-arachidonoyl glycerol, 2-(14,15-epoxyeicosatrienoyl) glycerol, and monoolein. Additionally, the following metabolites were elevated in the HCM group: corticosterone, 8-isoprostaglandin E2, LPE 18:3, 17α-hydroxypregnenolone, and 2-methoxyestrone; wogonin, sakuranin, and galangin (phenylacetone and polyketone classes); glycitein and genistein; palmitoyl ethanolamide, L-cystine, and taurine (organic acids and derivatives); and ceftibuten (organic heterocyclic compounds). Conversely, only L-arabinitol, adenine, and (R)-3-hydroxymyristic acid showed reduced levels.

Metabolic pathway enrichment was conducted for differential metabolites with serum FC > 1.2 or FC < 0.833 in menopausal women using the MetaboAnalyst website. The KEGG database was selected to screen for differential metabolic pathways based on the criteria of Pathway impact > 0.1 and *p* < 0.05, highlighting significant differences between the HCM and CM groups at baseline. The metabolic pathway identified at baseline was folate biosynthesis (*p* = 0.02). After 6 months of intervention, significantly different metabolic pathways between the HCM and CM groups primarily included pentose and glucuronate interconversions (*p* = 0.01). After 12 months of intervention, significant pathways included steroid hormone biosynthesis (*p* = 0.02) and prion diseases (*p* = 0.035) (Figure 5B). The significantly different metabolic pathways in the CM group after 6 and 12 months of intervention were mainly associated with glycerophospholipid metabolism (*p* = 0.015) and biotin metabolism (*p* = 0.03). In the HCM group, glycerophospholipid metabolism (*p* = 0.015), folate biosynthesis (*p* = 0.03), and arachidonic acid metabolism (*p* < 0.01) were observed. Therefore, glycerophospholipid metabolism appears to be a common metabolic pathway in both intervention groups, while arachidonic acid metabolism is specific to the high-calcium dairy interventions. Table 6 presents the metabolites primarily involved in arachidonic acid metabolism.

**Table 6:** Serum signature differential metabolites in the arachidonic acid metabolic pathway.

Metabolite name	Kegg name	0 m	12 m	AUC^1^
Prostaglandin D2	PGD2	296,050,146	484844985.1	0.754
5-OxoETE	5-OxoETE	2,656,648,260	4,579,503,628	0.728
12(S)-HETE	12(S)-HETE	55443468.9	101393102.7	0.757
Thromboxane B2	TXB2	88671676.1	154767018.7	0.724
Lipoxin B4	LXB4	222,405,089	363058588.1	0.730
16(R)-HETE	16(R)-HETE	50693496.5	87550647.18	0.681
Prostaglandin G2	PGG2	167,611,526	276772171.72	0.699
6-Keto-prostaglandin f1alpha	6-Keto-PGF1a	238,486,896	394209329.1	0.675

0 m, pre-intervention; 12 m, 12 months of intervention. ^1^The AUC values are interpreted as follows: an AUC of 0.5–0.7 indicates less predictive power, 0.7–0.9 suggests some predictive accuracy, and 0.9 and above denotes high predictive accuracy.

### 3.6 Fecal metabolome

**Figure 6:**
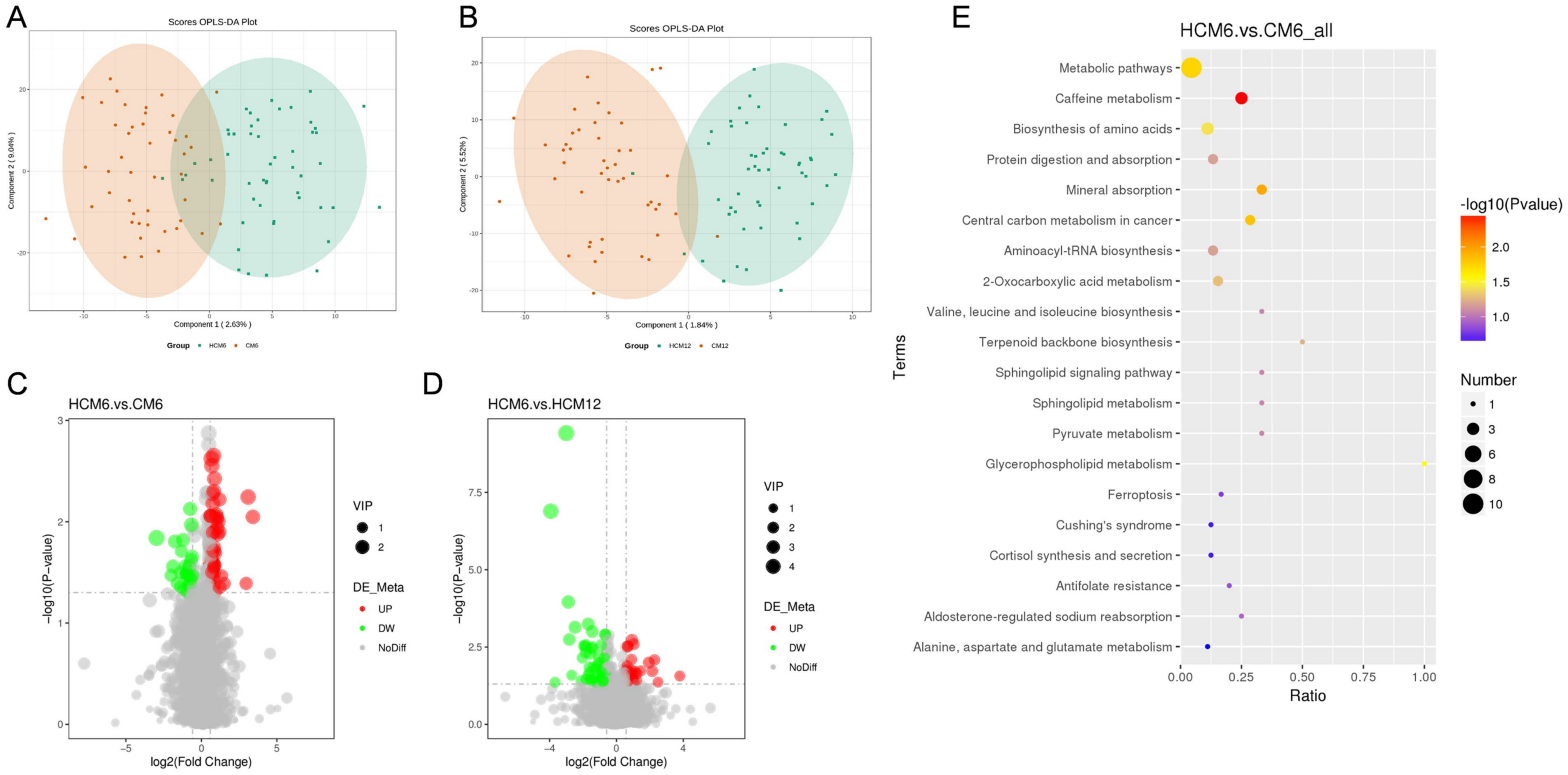
Differential fecal metabolites and pathways in the high-calcium milk and control milk groups. **(A,B)** PLS-DA plots of the high-calcium milk and control milk groups at 6 and 12 months after intervention. **(C,D)** Volcano plots of different metabolites in the high-calcium milk and the control milk groups at 6 months and 12 months after intervention. Each point in the plots represents a metabolite, with significantly upregulated metabolites indicated by red dots and significantly downregulated metabolites by green dots. **(E)** KEGG pathway enrichment analysis in the high-calcium milk and control milk groups after 6 months of intervention. The color of the points represents the *p*-values of the hypergeometric test, and the sizes of the dots represent the number of differential metabolites in the corresponding pathways.

PLS-DA was used to identify metabolite variability and account for individual differences in body metabolism. A PLS-DA model was constructed to assess the explanatory rate (R^2^) and predictive power (Q^2^) of the model. The results showed that metabolic changes in the HCM group were significantly different from those in the CM group over time, and the sample points were better clustered using the PLS-DA model (Figures 6A,B). Overfitting in the PLS-DA model was controlled using seven rounds of cross-validation based on the R^2^ and Q^2^ values, along with a rapid prototype test that involved 200 replications. These results showed that the PLS-DA model was stable and reliable for screening differential metabolites.

Analysis of differential metabolites in the HCM and CM groups across various intervention periods identified a total of 143 significant differential metabolites, with 100 upregulated and 43 downregulated. After 6 months of intervention, 74 significant differential metabolites were identified, with 47 upregulated and 27 downregulated. After 12 months of intervention, 43 significant differential metabolites were identified, with 27 upregulated and 16 downregulated. Volcano plots (Figures 6C,D) were generated based on the differential metabolites from each comparative group. The identified differential metabolites were functionally and taxonomically annotated using KEGG, indicating that these metabolites were mainly concentrated in the following pathways: global and overview maps, amino acid metabolism, lipid metabolism, digestive system, metabolism of cofactors and vitamins, endocrine system, nucleotide metabolism, and carbohydrate metabolism.

Metabolic pathways enrichment was performed for differential metabolites in fecal samples (FC > 1.2 or FC < 0.833) from menopausal women using the MetaboAnalyst website, with KEGG selected as the database to screen for differential metabolic pathways based on the criteria of Pathway impact > 0.1, and *p* < 0.05 (Figure 6E). At the 6-month intervention point, significant differential metabolic pathways between the HCM and CM groups included caffeine metabolism (*p* = 0.004), mineral absorption (*p* = 0.011), central carbon metabolism in cancer (*p* = 0.015), metabolic pathways (*p* = 0.018), glycerophospholipid metabolism (*p* = 0.030) and amino acids biosynthesis (*p* = 0.037). At the 12-month intervention point, differential metabolic pathways between the HCM and CM groups included ascorbate and aldarate metabolism, metabolic pathways, pantothenate and CoA biosynthesis, caffeine metabolism, pyrimidine metabolism, tyrosine metabolism, and purine metabolism. However, none of these differences reached significant levels.

### 3.7 Joint analysis

**Figure 7:**
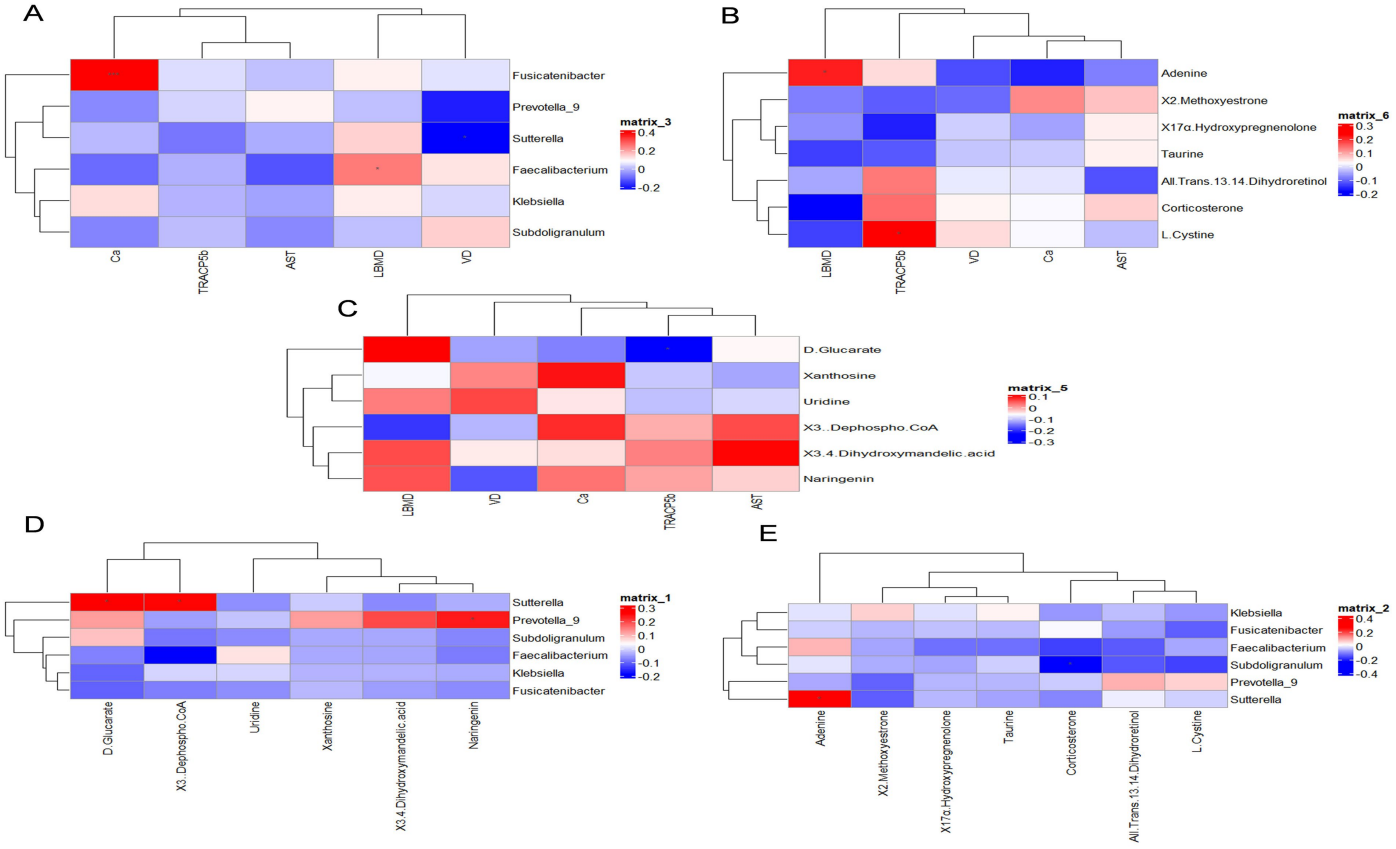
Correlation analysis. **(A)** Bone biochemicals correlated with key bacterial genera. **(B)** Bone biochemicals correlated with serum differential metabolites. **(C)** Bone biochemicals correlated with fecal differential metabolites. **(D)** Key bacterial genera correlated with serum differential metabolites. **(E)** Key bacterial genera correlated with fecal differential metabolites. Red denotes a positive correlation, while blue indicates a negative correlation.

To further explore the beneficial or detrimental relationships among gut microbes, serum, and fecal metabolites, as well as the mechanisms of dairy intervention in bone quality, we selected results after 12 months of intervention. Bone biochemical indices were analyzed in relation to key bacterial genera and representative serum and fecal metabolites using Spearman correlation analysis between the HCM and CM groups (Figures 7A–C). Our findings revealed that L1-4BMD was significantly and positively correlated with *Faecalibacterium* (*p* = 0.016) and adenine (*p* = 0.04). Calcium levels were significantly and positively correlated with *Fusicatenibacter* (*p* < 0.001), while 25(OH)D was significantly and negatively correlated with *Sutterella* (*p* = 0.028). Additionally, TRACP-5b was significantly negatively correlated with D-glucarate (*p* = 0.026) but significantly positively correlated with *L-cysteine* (*p* = 0.023). Subsequently, Spearman’s correlation analyses (Figures 7D,E) of key genera with representative serum and fecal metabolites were performed to assess the association between gut microbiota and metabolites. The results showed that *Prevotella_9* was significantly positively correlated with naringenin (*p* = 0.022), while *Subdoligranulum* was significantly negatively correlated with corticosterone (*p* = 0.025). Additionally, *Sutterella* was significantly negatively correlated with D-glucarate (*p* = 0.012), while it showed a significant positive correlation with 3-dephospho-CoA (*p* = 0.013) and adenine (*p* = 0.011). These results suggest that high-calcium milk may exert anti-osteoporotic effects through mechanisms involving cysteine and methionine metabolism, uric acid metabolism, metabolic pathways, pantothenate synthesis, and CoA biosynthesis.

## 4 Discussion

After middle age, BMD declines at a rate of 0.7–1.0% annually, and in postmenopausal women, this rate is three times higher than in premenopausal women (17–19). Studies have shown that postmenopausal women can reduce their bone loss by 1.0% per year by consuming calcium supplements. Numerous randomized controlled trials have confirmed that calcium supplementation, alone or combined with vitamin D, can reduce both bone loss and the incidence of osteoporosis (20). Dairy products are a primary source of nutrient-rich diets (21). In this study, we increased the amount of calcium derived from dairy products using plain low-temperature fresh milk with additional vitamin D and CPP as a potential intervention for PMO (22). CPP is a biologically active peptide derived from cow’s milk casein through biotechnology. Adding CPP helps maintain calcium in a soluble form within the intestinal tract, effectively promoting calcium absorption and utilization. Additionally, when CPP is incorporated into food products, it offers the advantages of high efficiency and low volume, while preserving the product’s original flavor and texture (23). A 1-year randomized controlled trial showed that adding 400 mL/day of either high-calcium or regular milk to the diet improved health and bone density, with high-calcium milk being more effective at increasing lumbar spine BMD and slowing or halting bone loss in the left hip and left femoral neck. These findings are consistent with a report by Postmenopausal Health, which conducted a 1-year clinical trial to examine the effects of dairy products fortified with calcium and vitamin D3 on BMD at various skeletal sites. They reported a 2.0% increase in lumbar spine BMD in the dairy group compared to other groups (95% confidence interval: 0.5–3.5); however, this difference was not statistically significant. The study also found that an integrated approach combining dietary intervention with fortified dairy consumption over 12 months led to favorable changes in pelvic, spinal, and whole-body BMD in postmenopausal women, although no changes were observed in quantitative ultrasound parameters of the calf bones (11). However, some data suggest that calcium and vitamin D supplementation may not affect bone density. One study found that higher dairy intake was associated with higher hip BMD in men but not in women aged 60 or older (24). A literature review further concluded that higher milk or dairy intake had no significant benefit for bone mass or fracture risk in women over 50, although this effect was not observed in women under 30 (25). Previous studies have also shown that the initial increase in bone density from calcium supplementation during the first or second year may not be sustained with continued treatment (26). In our study, during the first 6 months of high-calcium milk consumption, the bone remodeling space could absorb additional calcium, resulting in increased bone density at all sites. However, once this space was filled, bone density did not continue to increase significantly, and the effect gradually diminished despite continued high-calcium milk consumption. This differs somewhat from previous reports, which noted that reductions in bone loss with calcium supplementation primarily occurred in the first year (27), with no lasting density benefits at any skeletal site 2 years after the trial ended (28).

Studies have shown that the modulatory effect of high-calcium milk on bone health in menopausal women occurs after vitamin D is converted to its biologically active form 1,25-(OH)_2_D_3_, which modulates bone health in this population (29). Vitamin D status may be further compromised with age due to a decline in the skin’s ability to produce cholecalciferol, reduced liver or kidney’s hydroxylation of vitamin D to its metabolically active form, or as dairy intake or intestinal absorption of vitamin D decreases (30). We observed a significant increase in blood calcium levels in postmenopausal women in both the HCM and CM groups at 6 months after the intervention; however, high-calcium milk significantly improved vitamin D deficiency compared to the CM. According to the International Osteoporosis Foundation and National Osteoporosis Foundation guidelines, a 25(OH)D level of ≥20 ng/mL is considered adequate, and ≥30 ng/mL is the ideal level. The 25(OH)D level in the high-calcium milk group was closer to the ideal level after the intervention, suggesting that it significantly improved vitamin D status, promoted calcium absorption, reduced bone loss, and lowered fracture risk. Conversely, the enhancement effect of the regular milk group was limited. Therefore, we hypothesize that only a higher intake of calcium and vitamin D could increase and maintain BMD, thereby reducing the risk of hip fracture. However, recent data on the effects of vitamin D on BMD are inconsistent. A meta-analysis (31) and a randomized controlled trial (32) concluded that no evidence of an overall benefit of vitamin D supplementation was observed on BMD, although a small but relevant effect was observed on calcium absorption. Therefore, it remains unclear whether the effect of vitamin D on calcium absorption is sufficient to translate into a beneficial effect on BMD, particularly in older women with calcium deficiency, as represented in a clinical trial (32).

We explored the relationship and intrinsic mechanisms regulating gut microbiota and bone metabolism in patients with osteoporosis by monitoring changes in gut flora and metabolic profiles in menopausal women following high-calcium milk interventions, using 16S rRNA sequencing, and untargeted metabolomics. Our main findings indicate that high-calcium milk significantly affected the *β*-diversity of intestinal microbial structures in menopausal women. Additionally, the high-calcium milk group showed a significant increase in the abundance of several genera at 6 and 12 months of the intervention. L1–4BMD was significantly and positively correlated with *Clostridium pellucidum* and *Faecalibacterium*. Previous studies have also shown that osteoporosis and a higher risk of developing osteoporosis are associated with increased numbers of *Phascolarctobacterium*, *Actinobacillus*, *Blautia*, *Oscillospira*, *Bacteroides*, Ruminococcaceae, *Collinsella*, and Veillonellaceae (33). *M. anisopliae* is the main vitamin K-synthesizing bacterium in the human gut. It may prevent or treat osteoporosis by increasing BMD. Additionally, metabolites secreted by various anabolic bacilli are the main primary sources of SCFAs in the human gut—primarily acetic and propionic acids—which help maintain homeostasis and inhibit the release of proinflammatory cytokines by neutrophils and macrophages (34). The Trichosporon family is among the most abundant fungal taxa in the human gut, and recent clinical studies have shown that its abundance is positively correlated with bone density (35). *Oscillibacter* and *Faecalibacterium* are dominant SCFA-producing genera, particularly of butyrate, which is important for various aspects of human function and health (36). *Subdoligranula* has been shown to be a protective factor against BMD loss in individuals aged 45–60 and is known to produce SCFA butyric acid (37). Several studies have highlighted the immunomodulatory capacity of SCFAs (38). Since immune activation is closely related to bone homeostasis (39, 40), we hypothesize that gut microbes digest milk to produce SCFAs—particularly propionic acid and butyric acid—which help maintain bone homeostasis and inhibit bone resorption, thereby establishing a direct mechanistic link between the gut microbiota and bone health (41). Mechanistic studies have shown that propionate (C3) and butyrate (C4) induce metabolic reprogramming in osteoclasts, leading to enhanced glycolysis and reduced oxidative phosphorylation, which downregulates important genes in osteoclasts, such as *TRAF6* and *NFATc1* (42). However, only a few studies have explored the role of gut microbiota in bone homeostasis; these studies, conducted on antibiotic-treated mice, have reported conflicting results (43–45). Therefore, further studies are needed to elucidate whether therapeutic SCFA supplementation or increased endogenous SCFA production through dairy products can effectively balance osteoclast activity and inhibit bone resorption.

Furthermore, our metabolic pathway enrichment analysis of differential metabolites based on untargeted metabolomics using the KEGG database showed that high-calcium milk may improve bone quality through pathways related to steroid hormone biosynthesis, amino acid biosynthesis, and glycerophospholipid metabolism. A significant positive correlation between L1–4BMD and adenine was found using Spearman’s correlation analysis. This finding suggests that adenine nucleotides may play a role in bone metabolism, especially in maintaining or improving bone mineral density. However, it is important to emphasize that correlation analysis only reveals associations between variables and does not prove causality. Therefore, although this result is enlightening, further experimental validation is needed to clarify the specific mechanism of action of adenine nucleotides in bone metabolism. Results from our previous animal experiments also showed that ovariectomized rats could modulate gut microbiota composition and affect bone metabolism through pathways associated with steroid hormone biosynthesis (22). The first step in steroid hormone synthesis produces pregnenolone. Pregnenolone is catalyzed by the enzyme 3β-hydroxysteroid dehydrogenase into progesterone, which is then hydroxylated at position 17, enters the glucocorticoid pathway, and is further converted into androgens, eventually leading to A-ring aromatization to form estrogens (46). Estradiol has been shown to play an important role in women’s bone health by preventing rapid bone loss due to estrogen deficiency in the early stages of menopause (47). Methionine, an essential amino acid, is crucial for the synthesis of proteins and various functionally important biomolecules in the body. In recent years, studies in various model organisms have found that limiting dietary intake of methionine can prolong lifespan, improve metabolic health, and delay or ameliorate the onset and progression of many chronic age-related diseases (48). The metabolites involved in the glycerophospholipid metabolic pathway are predominantly lipids. Recent findings indicate that many diseases associated with lipid metabolism disorders, such as non-alcoholic fatty liver disease, atherosclerosis, obesity, and menopause, have been linked to the osteoporotic phenotype. Clinically, these lipid metabolism disorders have been associated with changes in osteoporosis-related indicators, including BMD and bone mass (49). Additionally, our serum metabolic analysis showed that the effects of high-calcium milk interventions may be closely related to the arachidonic acid metabolism pathway. Previous reports indicate that arachidonic acid and its metabolites mediate the synthesis of osteoblasts and osteoclasts, thereby influencing the development of osteoporosis (16).

**Figure 8:**
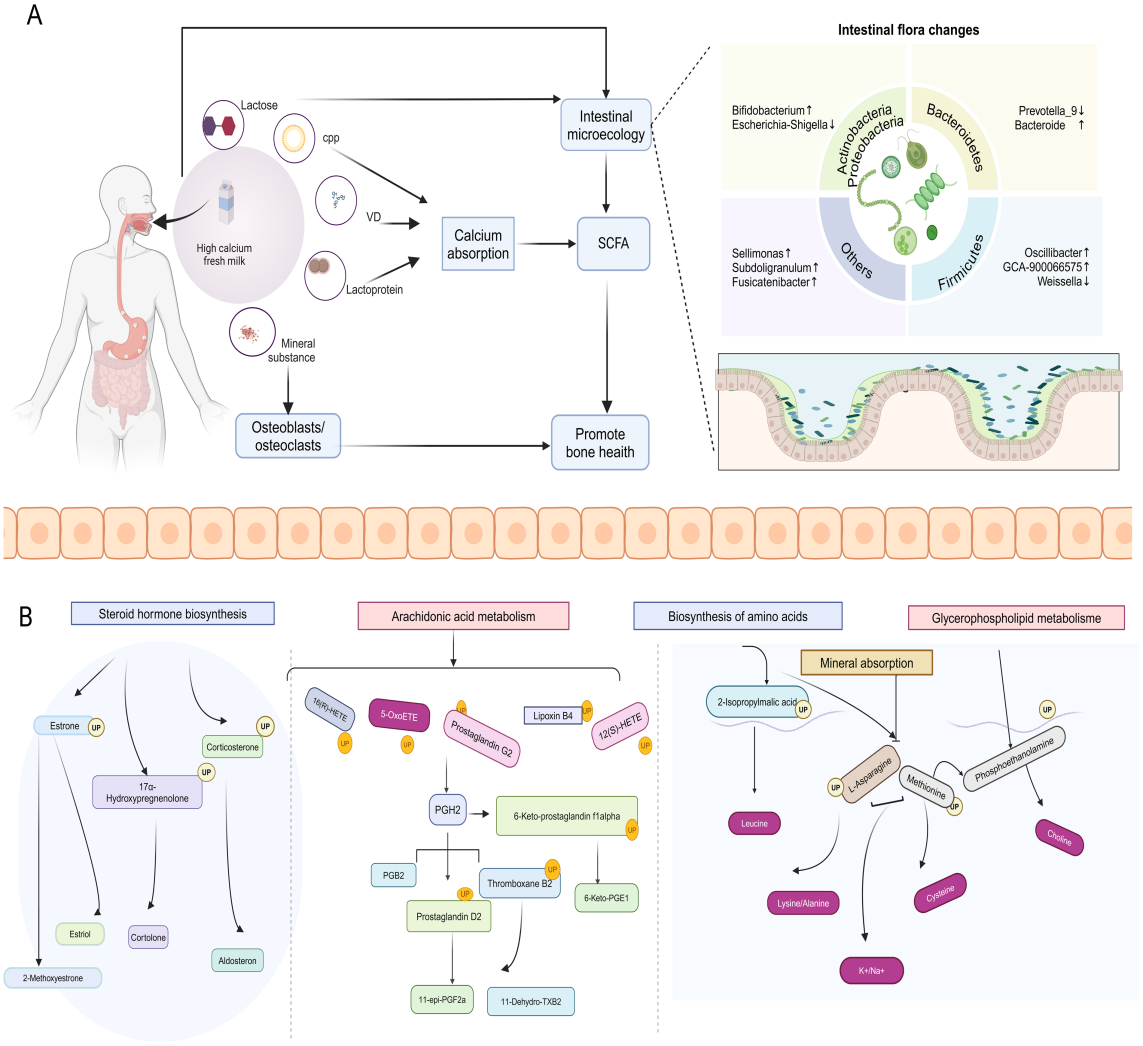
Potential mechanisms by which high-calcium milk affects bone metabolism. **(A)** Effects of high-calcium fresh milk on bone metabolism and intestinal flora in postmenopausal osteoporotic women. **(B)** Main pathways through which high-calcium fresh milk affects bone metabolism in postmenopausal osteoporotic women.

Our results suggest that high-calcium milk relies on calcium, vitamin D, CPP, and other nutrients that directly affect bone metabolism. Moreover, analysis of the experimental results revealed significant changes in serum calcium and vitamin D levels, bone metabolism markers, including AST, TRACP-5b, and PINP, and changes in the composition of the intestinal flora and serum and fecal metabolites before and after the intervention in the HCM group. Additionally, a significant correlation exists between BMD, the intestinal flora, and the metabolites. These findings suggest that high-calcium milk influences bone metabolism primarily through its nutrient content and by regulating intestinal flora and metabolites, thereby effectively promoting bone health (Figure 8).

However, some limitations to this study exist that may affect the interpretation and generalization of the results. First, the absence of a non-intervention control group limits the ability to fully assess the effects of the high-calcium milk intervention, particularly regarding its long-term benefits in preventing osteoporotic fractures, although ethical considerations played an important role in this design choice. Second, the lack of comparison between high-calcium fresh milk and room-temperature/long shelf-life milk made it difficult to clarify whether the observed benefits stemmed from calcium/vitamin D fortification or milk freshness, which made the attribution of the intervention effect less precise. Third, the 1-year follow-up period, while sufficient for observing short-term effects, was insufficient to fully assess the potential for long-term fracture prevention; therefore, extended follow-up is essential to fully assess the clinical significance of the intervention. Additionally, the study was conducted in a single geographic region, which may limit the generalizability of the findings. Regional differences in dietary habits, lifestyles, and environmental factors may have an impact on the results; therefore, a multicenter design should be considered for future studies to improve the extrapolation of results. Finally, although participants’ dietary habits were assessed at baseline and during monthly follow-ups, it was not possible to completely exclude confounding from other sources of calcium. Stricter dietary control or detailed record keeping may have contributed to a more accurate assessment of the intervention effect. These limitations suggest the need for caution in interpreting the study’s findings and emphasize the importance of improved designs in future studies to more comprehensively assess the potential benefits of high-calcium milk in the prevention of osteoporotic fractures.

In summary, this 1-year randomized controlled trial involving community-dwelling menopausal women with an average age of 61.2 ± 4.90 years demonstrated that dietary supplementation with 400 mL/day (approximately two cups) of milk fortified with calcium and vitamin D effectively increased lumbar spine BMD, halted or slowed bone loss in the left hip and left femoral neck, and raised vitamin D and phosphorus levels in postmenopausal women. Additionally, this supplement modulated bone formation markers and gut flora and has been linked to improved bone density in postmenopausal women. Our findings suggest that addressing the ecological dysregulation of gut steroid hormone biosynthesis and arachidonic acid metabolism may represent potential targets for osteoporosis interventions. Importantly, the fortified milk was well-tolerated by menopausal women and did not result in increased dietary fat (including saturated fat) intake or significant weight gain. Milk is nutrient-dense and contains various essential nutrients for bone health; therefore, milk fortified with calcium and vitamin D may offer a simple, nutritionally sound, and cost-effective approach to reducing the future health and economic burden of osteoporosis in menopausal women. Future studies should determine the optimal levels of calcium and vitamin D fortification in dairy products and explore their relationship with bone health and clinical outcomes, such as fractures, in other populations.

## Data Availability

The raw data supporting the conclusions of this article will be made available by the authors, without undue reservation.
